# Cognitive and non-cognitive variables influencing age-related effect of mind wandering across the adult life span

**DOI:** 10.1007/s10433-021-00637-3

**Published:** 2021-07-14

**Authors:** Erika Borella, Michela Zavagnin, Lucia Ronconi, Rossana De Beni

**Affiliations:** Department of General Psychology, University of Padova, Padova, Italy

**Keywords:** Mind wandering, Life span, Working memory, Attention, Inhibition

## Abstract

This study aimed to assess the effects of aging on mind wandering (MW) using a sustained attention to response task (SART) with a low cognitive demand. All task-unrelated thoughts (TUTs) and the subcategory of stimulus-independent thoughts (SITUTs) were examined across the adult life span. The relationship between MW, cognitive variables (attention, inhibition, working memory), and non-cognitive variables (mindfulness, psychological well-being, and anxiety) was investigated. The sample included 210 healthy participants from 20 to 89 years old. The overall results showed few or no age-related changes in both TUTs and SITUTs. Path analyses revealed that the effect of age on both TUTs and SITUTs was only indirect and mediated by attentional resources, as well as by some aspects of psychological well-being (i.e., emotional competence), which had a direct effect, however. These findings raise doubts about any age-related differences between young and older adults’ MW. Changes in MW across the adult life span are thus discussed along with the method and tasks used to assess it and different variables affecting it.

## Introduction

In everyday life, our attention is not always on the here and now. It fluctuates between mental content from both intrinsic and extrinsic sources. A long tradition of research has confirmed that people’s attention is continually shifting from a current train of thought (often an external task) to mental content generated by the individual rather than by the environment only (Smallwood and Schooler 2015). This complex phenomenon is called mind wandering (MW). It can be intentional or unintentional, and it can thus involve a greater or lesser awareness and disengagement from the external stimuli being processed at the time. Cheyne et al. (2009) clearly explained the interaction between MW intentionality and engagement in the task by means of a model of attentional engagement/disengagement involving three states. The first involves a transient disengagement of attention from the task: individuals are often aware of this and can decide whether to suppress or indulge their MW. In the second state, their attention diminishes, but they continue to produce well-practiced automated responses; they are less aware of their MW, but may be alerted to it when they make mistakes in the task at hand. In the third state, they may be so absorbed in their MW that they become unresponsive to anything else and consequently fail to complete their task.

The content of MW also varies: task-unrelated thoughts (TUTs) occurring during MW experiences are often self-generated daydreams or worries about life called stimulus-independent TUTs (SITUTs). These SITUTs possess the core characteristics of MW: the content is unrelated to the activity underway (Giambra 1989, 1995), and they are decoupled from any surrounding stimuli (Antrobus 1968; Teasdale et al. 1995), so they are sometimes identified with MW itself. Distractions occurring while performing a task can originate not only from SITUTs, however, but also from sensory perceptions/sensations or external distractions (EDs), or from thoughts (considerations or worries) related to the ongoing task (task-related interferences [TRIs]) (Stawarczyk et al. 2011).

Most of the research on MW has examined this phenomenon in younger adults, but interest has recently increased in studying MW in aging (see Table 1) because of the important consequences for older adults’ well-being and decision-making (Smallwood et al. 2009). What little we know, as yet, about the age-related effects in MW across the adult life span, comes from two studies. Using retrospective reports, Giambra (1989) examined TUT frequency in participants from 17 to 92 years. However, because of small sample sizes and differences at a methodological level, the results were inconsistent across different experiments run (see Table 1), and thus, they cannot be considered exhaustive. Seli et al. (2017) used a specific MW questionnaire to examine the relation between intentional and unintentional MW in daily life in a large sample of participants ranging from 16 to 82 years of age; they found that only intentional MW decreased with age. Questionnaires rely, however, on respondents’ ability to recall the thoughts they had while they were previously completing an activity. As this may give rise to a bias, without capturing the real frequency of MW, it is worth seeking any age-related changes in MW by: (i) examining adults of all ages, even the oldest-old, to obtain a clearer picture spanning the whole adult life span, and (ii) using a probe-caught method and sustained attention to response tasks (SARTs), rather than retrospective questionnaires. SARTs are widely used in MW research, in various versions that manipulate their difficulty by modifying the frequency of the targets, the intra-stimulus interval (ISI), and the total duration. The less demanding SART version (e.g., with fewer targets, or a longer ISI) has been recommended as the most suitable for studying MW in the elderly population (Giambra, 1989; Jordao et al. 2019).

**Table 1:** Summary of the characteristics and results of MW studies that included older adults (published in peer-reviewed journals)

Authors	Sample size	Tasks	Sampling method	Results
Giambra (1989)	Ex. 1: 108 individuals (aged 22- 89)	Ex. 1 and 2: Vigilance task. ~ 62 min duration. ISI 0.5 s; target 0.6%	Ex. 1: self-caught	Ex. 1: No significant age effects
	Ex. 2: 117 individuals (aged 17–92)		Ex. 2: probe-caught	Ex. 2: TUTs decreased from 50–59 year-old age group onwards
	Ex. 3: 187 individuals (aged 17–92)	Ex. 3 and 4: Vigilance task. ~ 25 min duration. Two blocks a) ISI 10 s.; target 42%; b) ISI 20 s.; target 7.5%	Ex. 3: self-caught	Ex. 3 and 4: TUTs decreased from 60–69 year-old age group onwards
	Ex. 4: 51 individuals (aged 17–69)		Ex. 4: probe-caught	
	Ex. 5: 20 YAs (aged 17–39), 20 OAs (aged 60–89)	Ex. 5: and 2 Vigilance task. ~ 198 min duration. 5 blocks ISI from 2 to 32 s.; target 30% and 10%	Ex. 5: probe-caught	Ex. 5: TUTs decreased with age
Einstein and McDaniel (1997)	24 YAs (aged 18–21),	Memory task. 675 words, the recall of which was tested nine times	Indirect measure of MW: number of words recalled	No significant age-related differences
	24 OAs (aged 60–78)			
Jackson and Balota (2012)	Ex. 1: 54 YAs (M = 19. SD = 0.9), 62 OAs (M = 77.3. SD = 6.9)	Ex. 1: SART. ~ 4 min duration. ISI 0.9 s.; target 11.1%	Ex. 1: probe-caught (1.7% of trial)	In all experiments YAs reported more TUTs (overall MW) than OAs
	Ex. 2 and 4: 32 YAs (M = 19.4. SD = 0.8), 38 OAs (M = 78.8. SD = 6.5)	Ex. 2: SART presented as in Ex. 1. but ~ 5 min duration	Ex. 2: probe-caught (4% of trial)	
	Ex. 3: 31 YAs (M = 20.9. SD = 1.4), 49 OAs (M = 76.3. SD = 6.4)	Ex. 3: SART presented as in Ex. 2. but ~ 10 min duration and 1.25 s. ISI	Ex. 3: probe-caught (4% of trial)	
		Ex. 4: Reading comprehension task. ~ 30 min duration	Ex. 4: self-caught and probe-caught	
Krawietz et al. (2012)	Ex. 1: 78 YAs (ages 18–22), 78 OAs (ages 58–87)	Ex. 1 and 2: Reading comprehension task	Ex. 1 and 2: Probe-caught (every 2–4 min)	Ex. 1 and 2: YAs reported more MW than OAs
	Ex. 2: 63 YAs (ages 17–22), 23 OAs (ages 62–86)			
Jakson et al. (2013)	Ex 1. 89 YAs (aged 18–30) 57 OAs (aged 50–70)	Ex 1and 2. SART ~ 14 min duration. ISI 1.25 s.; target random but more than 1.4%	Ex. 1 self-caught	In both experiments, YAs reported more MW than OAs
	Ex 2. 82 YAs (aged 18–30) 74 OAs (aged 50–73)		Ex 2. Probe-caught (14 probes, semi-randomized presentation)	
Maillet and Rajah (2013)	31 YAs (ages 18–32), 26 OAs (ages 60–76)	Two encoding tasks: 1) judging whether words are man-made/natural, 2) judging whether words are pleasant/neutral	Retrospective questionnaire measured TRIs and SITUTs	YAs reported more TRIs and SITUTs than OAs. Only YAs reported more TRIs than SITUTs during man-made/natural task and more SITUTs during pleasant/neutral task
McVay et al. (2013)	Ex. 1: 108 YAs (ages 18–28), 99 OAs (ages 60–75)	Ex. 1: Standard and vigilance SART. ~ 20 min duration. 900 ISI.; target 11%	Ex. 1: probe-caught (followed 60% of target)	Ex. 1: in both SART versions, YAs reported more TUTs than OAs
				Ex. 2: OAs reported fewer TUTs in all tasks and in 2-*back* task they reported more TRIs than YAs
	Ex. 2: 112 YAs (ages 18–29), 85 OAs (ages 60–75)	Ex. 2: 1, 2 and 3-*back*. ~ 25 min duration. 2500 ISI.; target 25%	Ex. 2: probe-caught (30 probes)	
Zavagnin et al. (2014)	20 YAs (ages 20–30),	Perceptual and Semantic SART. ~ 10 min duration. ISI 0.5 s.; target 16.3%	Probe-caught (24 probes)	YAs reported more TUTs than OAs or OOs
	20 OAs (ages 65–74),			
	19 OOs (ages 75–85)			
				OOs reported more TRIs and more TUTs in the semantic SART
Frank et al. (2015)	36 YAs (ages 18–25),	Reading comprehension task	Probe-caught and eye movements	OAs reported fewer TUTs and more TRIs than YAs
	40 OAs (ages 60–85)			
Fountain-Zaragoza et al. (2016)	75 OAs (ages 60–74)	Go/No-Go task: ~ 60 min duration, ISI 750 ms	Probe-caught (18 probes, after 22.5, 30 or 37.5 s) in both tasks	TUT rates were not associated with task performance
		Continuous Performance (CP) task: ~ 35 min		TUTs mediated the association between mindfulness and proactive control on the CP
Mallet and Schacter (2016)	30YAs (ages 18–34),	Incidental encoding task: ~ 30 min duration	Probe-caught (18 probes, after 22.5, 30 or 37.5 s	OAs reported fewer stimulus-independent thoughts and more stimulus-dependent thoughts than YAs
	30 OAs (ages 65–87)			
Jordano and Touron (2017)	30 YAs (ages18-25)	Operation span task with or without prior activation of the age stereotype (ST)	Probe-caught (9 probes, after ~ 120 s)	OAs reported fewer TUTs and more TRIs than YAs. ST-activated OAs did not report more reactive TRIs than OAs who were not ST-activated
	90 OAs (ages 60–75)	Dundee stress state questionnaire	Retrospective questionnaire	
Seli et al. 2017	Ex. 1 795 people (ages 16–82)	Ex. 1 Data collection in daily life	Ex.1 Intentional/Unintentional MW Questionnaire	Ex 1. Only deliberate MW was negatively related with age
	Ex. 2 29 YAs (ages 18–28) 27 OAs (aged 65–88)	Ex. 2 SART ~ 20 min duration. ISI 1,65 s	Ex. 2 Probe-caught (18 probes)	Ex 2. OAs reported fewer TUTs than YAs and both age groups reported more unintentional than intentional MW
Gyurkovics et al. (2018)	270 MA (ages 43–69)	SART. ~ 5 min duration. ISI 1.25 s.; target 11.1%	Probe-caught (5 probes appeared randomly)	MA adults reported more TUTs, especially tune outs (MW with awareness), than OAs, who in turn reported more than ADs
	282 OAs (ages 70–94)			
	77 ADs (ages 63–95)			
Maillet et al. (2018)	31 YAs (ages18-34)	Data collection in daily life (7 days) through an app-based platform for experience sampling data collection, that was downloaded to personal smartphone or tablet	Probe-caught (12 probes per day, random, at least 50 min)	OAs reported fewer TUTs and more TRIs than YAs. Older adults tended to have more pleasant, clear and interesting thoughts
	20 OAs (ages 66–77)			
Warden et al. (2019)	Ex 1. 21 YAs (ages 18–33) 19 OAs (ages 65–86)	Ex 1. Data collection in daily life (2-week diary)	Ex 1. Self-caught	Ex 1. No significant age-related differences. More involuntary than spontaneous thoughts
	Ex 2. 24 YAs (ages 18–38) 22 OAs (ages 67–90)	Ex 2. Data collection in daily life (10 h) using a tablet app	Ex 2. Probe-caught (30 probes, random between 15- to 25-min intervals)	Ex 2. No significant age-related differences. TRIs and SITUTs were more frequent than EDs, and more spontaneous than deliberate TUTs

*Ex* Experiment, *YA* younger adult, *MA* middle-aged participants, *OA* older adult (young-old adults), *OO* old-old adult, *AD* Alzheimer’s disease, *MW* Mind Wandering, *TUT* task-unrelated thought, *TRI* task-related interference, *SITUTs* thoughts or concerns about personal life and daydreams, *TRIs* task-related interference thoughts, *EDs* sensory perceptions/sensations or external distractions

Whatever the version of SART used, MW could be assessed by asking participants to report when they realize their mind is wandering while completing the task (self-caught method), or by presenting probes in response to which they classify the content of their immediately preceding thoughts (probe-caught method) (Smallwood & Schooler, 2006). The latter method was used here because it disrupts the natural frequency of TUTs less and does not require good self-monitoring ability (which often declines with aging).

### Role of cognitive and non-cognitive variables

The mechanisms behind MW have also been the object of study. According to the executive control failures × current concerns view, TUTs occur when the executive control fails to block automatically generated interfering thoughts adequately (McVay and Kane 2010). In accordance with this view, younger adults with more efficient working memory –WM– (high-performer) typically report fewer TUTs than poor performers, particularly during attention-demanding tasks (Mooneyham and Schooler 2013). Then, it would seem logical to expect older adults’ minds to wander more, given the known age-related decline in basic cognitive mechanisms such as inhibition and WM (Borella et al. 2008). The results reported in the literature (see Table 1) do not support this hypothesis, however; 20 out of the 24 experiments focusing on MW and aging found a decrease in TUTs with age—as also supported by the results of the meta-analysis conducted by Jordão et al. (2019)—while four identified no age-related differences.

Studies that focused on intrusive thoughts (Maillet and Schacter 2016), which can be considered particular types of TUT (they are unintentional and have a negative content), generated mixed findings, however. Some reported that these particular types of TUT—intrusive thoughts—declined with age (e.g., Berntsen et al. 2015); others found that they increased over time, but only in the oldest older adults (e.g., Stawski et al. 2011). This might indicate that the elderly experience more intrusive thoughts than younger adults, a trend that would be in line with studies showing that inhibitory processes follow a quadratic trend with aging (Borella et al. 2008). The existence of quantitative differences depending on the type of MW considered (TUTs overall, or intrusive thoughts alone) also seems to be corroborated by studies showing higher proportions of unintentional MW (Seli et al. 2017) in the elderly than in the young. Older adults therefore more often seem to match the second state of the Cheyne et al. attentional engagement/disengagement model (2009) and to be less aware of their TUTs than younger people. This would be confirmed by the age-related differences found between young and older adults found in the SART performance indices (i.e., considering variability in response times, wrong answers, and omission errors) (Zavagnin et al. 2014). So it may be that the seemingly paradoxical finding of a negative relation between MW and age is due to studies having focused on specific (and consequently different) aspects of MW.

Alternatively, Krawietz et al. (2012) have suggested that the decrease seen in TUTs with aging could be explained by the decoupling hypothesis (Smallwood and Schooler 2006), which predicts that individuals having fewer cognitive resources are less likely to indulge in TUTs. Older adults may therefore experience this effect because of the well-documented general decline in their processing resources with aging (e.g., Borella et al. 2011; Meneghetti et al. 2014). This hypothesis seems to be confirmed by the presence of more accentuated age-related changes in TUT frequency in older adults who have fewer cognitive resources (i.e., early-stage Alzheimer’s disease; Gyurkovics et al. 2018); it is unable, however, to explain why TUTs do not decrease proportionally as the difficulty of a task increases in older adults (Zavagnin et al. 2014) or why some studies have found that all categories of TUTs tend to decrease in older adults with the exception of TRIs, which increase instead (Jordano and Touron 2017).

To elucidate these inconsistencies, other aspects influencing MW in aging need to be considered. For example, some authors suggest that older adults produce more TUTs, but they tend to consider them a normal product of the mind, so they are less likely to report them (Giambra 1989). A study using eye movements demonstrated, however, that older adults report their TUTs as accurately as younger participants (Frank et al. 2015).

Another possibility proposed is that older adults allocate more of their resources to a task at hand than their younger counterparts because they are more conscientious (Jackson and Balota 2012), more interested in the task (Maillet and Rajah 2013), and more motivated (Seli et al. 2017), or because they find it more difficult. It is important to note, however, that age-related differences in MW are observed even when performance is at ceiling in both age groups (Giambra 1989), or when task difficulty is adapted to each subject (Krawietz et al. 2012). It has also been speculated that the laboratory setting, being more foreign to the elderly and more likely to arouse stereotypes, could prompt them to engage (concentrate) more on a task and also experience an increase in TRIs (Jordano and Touron 2017). That said, similar effects, involving an increase in stimuli-dependent thoughts (such as TRIs) and a reduction in SITUTs, have been found in everyday activities as well (Warden et al. 2019).

Another hypothesis postulates that older adults may have fewer concerns or goals, and may be less anxious about their everyday life, or have less “unfinished business” to do (see Giambra 1989): older adults’ greater focus on the present moment, or mindfulness (Splevins et al. 2009; Frank et al. 2015), and their generally more positive mood (Carstensen et al. 1999) may mediate the reduction in MW with aging. Such a result can be explained by age-related variations in emotional regulation and life goals (Carstensen and Charles 1998) in older adults. Numerous studies have further shown that mood—i.e., dysphoria and anxiety—has been found associated with more frequent TUTs in younger adults (e.g., Kane et al. 2007; McVay et al. 2009; Smallwood et al. 2007; 2009), as well as the simple experience of a sad mood or less happiness in both young (Killingsworth and Gilbert 2010; Poerio et al. 2013) and older adults (Maillet et al. 2018). This is because people who are sad or worried tend to ruminate and experience more negative automatic thoughts than people perceiving a sense of well-being (Smallwood et al. 2007).

Whatever the “hypotheses” advanced to explain MW, it is worth emphasizing that the heterogeneity of the studies conducted to date, in terms of methodological differences and sociodemographic factors relating to MW, can explain their discrepant findings regarding the effects of aging, as the Jordão et al. (2019) meta-analysis demonstrated. For instance, age-related effects may be influenced by different gender proportions in the age groups being compared (because women tend to report more frequent TUTs than men), or by the inclusion of old-old participants. Then, using less demanding tasks and probe-caught MW recording methods seems to give a more accurate picture of MW in aging, better distinguishing between various categories of TUTs. In fact, participants often misclassified TRIs (saying: "My attention is completely on-task"), failing to classify them as TUTs because they were task related. It was as if the TRI option was not among the probe response options (Robinson et al. 2019). On the other hand, a different trend was seen with aging in the occurrence of TRIs and EDs, as compared with SITUTs (see Maillet and Schacter 2016 for a review).

In light of the above considerations, we administered a version of the SART with a low cognitive demand (the perceptual SART, Zavagnin et al. 2014) with a high proportion of target stimuli to participants from 20 to 89 years old (equally distributed by age and gender). We used the probe-caught method, but also a retrospective questionnaire to collect further quantitative information on respondents’ reported awareness and intentionality of their TUTs during the test.

The age-related changes in various categories of TUTs (SITUTs, TRIs, EDs) were examined first. Then, we focused on analyzing the mechanisms underlying the MW episodes, considering the frequency of both TUTs as a whole (to be able to compare the results with studies that did not distinguish between types of TUTs) and SITUTs alone. To be more precise, while examining the effects of age on the frequency of TUTs overall and SITUTs alone across the adult life span, a complementary goal of the present study was to assess the joint role of processing resources (attention, WM, inhibitory control functions) and non-cognitive variables (mindfulness, anxiety, perceived well-being) in explaining variations in MW frequency.

Classical cognitive tests measuring WM (the Listening span test, which also gives an indication of inhibitory efficiency, or intrusion errors, i.e., Borella et al. 2008), inhibition (the Stroop color test, Trenerry et al. 1989), and attention (the D2 task, de Ribaupierre and Lecerf, 2006) were used, along with questionnaires assessing mindfulness (Mindful Attention Awareness Scale, Brown and Ryan 2003), anxiety (State-Trait Anxiety Inventory, Zotti et al. 1985), and psychological well-being (Psychological Well-Being Questionnaire, De Beni et al. 2008).

In line with previous studies (see Table 1), we expected to find that aging coincided with a decrease in TUT and SITUT frequency. The size of this decrease might be small, however, because the version of SART used is less demanding than other tasks used in the literature (e.g., the *n*-back). Age-related changes were expected, in terms of: i) a decrease in processing resources (WM, attention and inhibition, e.g., Borella et al. 2011; Delaloye et al. 2008; Cantarella et al. 2017) and in levels of anxiety (although lifetime patterns of anxiety remain a debated issue; Bryant et al. 2008) and ii) an increase in the levels of well-being and mindfulness (Frank et al. 2015).

Path analyses were also run to better capture how these variables interact. In line with the literature (e.g., Craik and Salthouse 2008), we expected age to have a direct relationship to cognitive and non-cognitive variables. Taking the executive control failures × current concerns view, we might expect a negative relationship between WM and TUT frequency (McVay and Kane 2010). In contrast, based on the decoupling hypothesis, we might find a direct and positive relationship between attentional resources and frequency of TUTs (Smallwood and Schooler 2006). Applying these two hypotheses (developed with young adults) to the whole adult life span, we might also expect age to have both direct and indirect effects on TUT frequency, mediated by WM or attentional resources. In particular, we might expect the age-related change (decrease) in WM to cause an increase in TUTs (as predicted by the control failures × current concerns hypothesis) or find that the age-related decrease in attentional resources induces a decrease in TUTs (as predicted by the decoupling hypothesis).

For the non-cognitive variables, in agreement with both the above-mentioned theories, we can expect psychological well-being, anxiety, and mindfulness to have a relationship with the amount of TUTs reported by participants (Maillet et al. 2018); in particular, the increase in psychological well-being and mindfulness with aging could mediate age-related effects on TUT frequency (Frank et al. 2015). We explore whether these effects might be similar for SITUTs (or more marked) because they are considered a core aspect of MW, which is less affected by classification errors (such as the misclassification of TRIs) or the greater distractibility of the elderly exposed to external stimuli.

We therefore explored the direct and indirect effects of all these factors on the frequency of TUTs (and SITUTs). The influence of age, as a mediator or otherwise, on TUTs (and SITUTs) was also examined.

## Method

### Participants

Our sample consisted of 210 participants^1^ (103 females and 107 males) from 20 to 89 years old. For each ten-year age bracket, there were 30 volunteers, all recruited by word of mouth. Universities of the third age and recreation centers (bowling clubs, older workers’ associations) were contacted to recruit people over 65 years old. Participants were all Italian-born residents of various Italian cities: the younger adults were students or workers; those over 65 were all community dwellers with no history of psychiatric or neurological disorders, diseases causing cognitive impairments, visual, auditory and/or motor impairments, or cognitive difficulties, as confirmed by the Short Portable Mental Status Questionnaire (SPMSQ) for assessing organic brain deficits in the elderly (see Pfeiffer 1975). The age groups did not differ in terms of participants’ years of formal education or, in line with the results of other Italian studies (i.e., Pezzuti et al. 2012), vocabulary score (Wechsler 1981) (see Table 2).

**Table 2:** Descriptive statistics (M and SD) for the measures of interest by age group

	Ages 20–29		Ages 30–39		Ages 40–49		Ages 50–59		Ages 60–69		Ages 70–79		Ages 80–89	
	N = 30		N = 30		N = 30		N = 30		N = 30		N = 30		N = 30	
	M	SD	M	SD	M	SD	M	SD	M	SD	M	SD	M	SD
*Characteristics of sample*														
Age	24.65	2.41	35.13	3.20	44.17	2.28	54.07	2.36	64.33	2.86	74.10	2.75	83.97	2.98
Education (years)	13.20	3.38	13.63	3.08	12.93	3.61	12.83	3.78	11.60	4.37	11.30	5.04	11.77	5.41
Vocabulary	47.23	7.33	48.97	7.00	49.53	7.07	48.03	0.91	49.37	8.06	45.67	10.32	46.30	8.09
*Cognitive resources*														
LST (correct recall)	29.00	4.88	27.63	4.70	27.03	5.56	25.43	5.35	22.90	5.82	19.43	4.99	16.90	6.23
D2 (correctly marked items)	131.80	28.02	127.93	28.82	115.70	28.22	104.00	31.73	91.60	29.60	73.93	26.84	58.97	26.18
Color Stroop Interference Index (RT)	0.66	0.23	0.67	0.24	0.76	0.25	0.88	0.31	1.01	0.42	1.16	0.67	1.02	0.49
LST proportion of intrusion errors	0.08	0.07	0.10	0.10	0.10	0.10	0.11	0.10	0.16	0.14	0.22	0.26	0.24	0.16
*Non-cognitive variables*														
PS-WBQ	30.87	3.82	31.67	4.89	30.93	5.30	32.33	4.68	32.43	5.79	33.33	6.72	36.07	5.76
CS-WBQ	24.53	3.38	24.63	3.44	24.87	4.14	25.77	3.76	25.90	4.59	24.20	4.94	25.33	4.62
EC-WBQ	26.70	3.69	28.23	4.29	28.30	5.09	27.90	4.39	29.00	4.94	28.80	5.37	29.83	5.96
STAI-X	39.63	8.48	36.30	6.07	39.63	7.54	39.00	6.81	36.50	7.89	43.27	8.30	39.83	7.72
MAAS	40.10	7.58	35.17	8.62	37.40	9.94	35.90	9.96	32.40	8.34	36.33	8.51	34.37	9.69

*LST* Listening Span Test, *WBQ* Well-Being Questionnaire, *SP-WBQ* Well-Being Questionnaire, personal satisfaction subscale, *CS-WBQ* Well-Being Questionnaire, coping strategies subscale, *EC-WBQ* Well-Being Questionnaire, emotional competence subscale, *STAI* State-Trait Anxiety Inventory, *MAAS* Mindful Attention Awareness Scale, *SART* Sustained Attention to Response Task

### Materials and Methods

#### Mind wandering

Sustained Attention to Response Task (SART) (Zavagnin et al. 2014)—This is a go/no-go task involving 172 stimuli (144 non-target, 28 target) consisting of five strings of “X” (target stimuli) and five of “O” (non-target stimuli) arranged in blocks of 5, 6 or 7 strings with 0, 1, or 2 targets. The blocks and the strings they contained were presented in random order. Based on Giambra’s recommendations (1995) and comments from Jackson and Balota (2012) regarding the need to present stimuli more slowly if a task is also intended for older people, each string was presented in the middle of the screen for 2000 ms, with an inter-stimulus interval (ISI) of 2000 ms. The screen was black during the ISI. Participants sat about 50 cm away from the screen. The experiment was conducted using the E-Prime software.

Participants were asked to press a green button whenever a non-target stimulus (81%) appeared. The D-prime index was calculated from the percentages of correct hits and correct rejections. Every 22–30 s, the following message appeared: “If your attention was completely on the task, press the RED button. If you had other thoughts, press the GREEN button, and select what type of thoughts you had from among those listed”. Participants were instructed to consider whether their mind had been wandering immediately before the probe appeared. They were also asked to classify their thoughts as: i) on-task; ii) SITUTs: thoughts or worries about personal life, and daydreams; iii) TRIs: task-related interferences; iv) EDs: sensory perceptions/sensations or external distractions; v) not known.

After completing the SART, participants were asked to indicate on a Likert scale how difficult it had been to classify their MW episodes (from 0 = very difficult to 6 = very easy) and how accurately they felt they had done so (0 = not at all, 6 = perfectly).

The last test was a 12-item debriefing questionnaire assessing the perceived frequency of various types of thoughts coming to mind while performing a task and participants’ intentionality regarding the presence of these thoughts. Participants were asked to indicate on a 5-point Likert scale how often they experienced the situations described (from 0–20% to 90–100% of the time). Some examples of the items to be rated are: "Fantasizing or daydreaming" (classified as SITUTs); "Being distracted by environmental stimuli (e.g., a noise)” (EDs); "Worrying about the results you would get in this test" (TRIs); "Deliberately allowing your mind to think of something else” (intentional MW); "Being surprised that, for a few seconds, your mind was thinking about something else" (unintentional MW).

#### Cognitive measures

##### Working memory

Listening span test, LST (Borella et al. 2007). The task consists of an increasing number of simple sentences grouped into 2 sets. Twenty sentences are presented for each set (for a total of 40 sentences), with two series of two, three, four, five and six sentences; each sentence is separated from the next by an interval of 1.5 s. Participants are instructed to listen carefully to each sentence, judge its plausibility (say whether it is true or false) and retain the last word. At the end of the series of sentences, participants are asked to recall orally all the final words that were presented in the series. Two training trials precede the task.

The total number of last words recalled correctly and in the correct order during the whole test was considered as the measure of the participant’s WM capacity. The number of intrusion errors (see below) was also computed. The reliability of the test was good (split-half procedure with Spearman–Brown correction = 0.86).

### Attention

D2 task (de Ribaupierre and Lecerf 2006). The version of this paper-and-pencil cancelation test used here was adapted from Brickenkamp (1998). It consists of 10 trials. In each trial, there is a row of 47 “p” and “d” characters arranged adjacent to one another on a sheet of A4 paper. The characters can have from one to four dashes placed separately or in pairs above and/or below each letter. Participants were allowed 20 s to scan each line (after which time they were asked to go on to the next row) and cross out the letter “d” with two dashes. No pauses were allowed between trials. The score used in the present study was the total number of characters correctly crossed out on each of the last nine rows, as in de Ribaupierre and Lecerf (2006). The reliability of the test was good (Cronbach’s α on the correctly processed = 0.98).

#### Inhibition

Stroop color task (adapted from Trenerry et al. 1989). The task consists of 16 cards that list: 15 “X” characters printed in different colors (Neutral-Control 1 condition), 15 names of colors printed in incongruent colors (Incongruent condition), 15 names of colors printed in congruent colors (Congruent condition), and 15 color patches (Neutral-Control 2 condition). There were 4 cards for each condition. Participants were asked to name the color of each stimulus and process the stimuli as fast as possible while also being as accurate as possible.

To adjust for baseline individual differences (see Ludwig et al. 2010), the interference effect was calculated as the relative difference in the time taken to complete each card, as follows: [(incongruent condition – control 2 condition)/control condition]. A higher score thus implied greater difficulty in controlling the prepotent response in the incongruent condition. The reliability of the task was acceptable/good (split-half procedure with Spearman–Brown correction: control condition 1 = 0.92, incongruent condition = 0.96, congruent condition = 0.50, control condition 2 = 0.94).

Intrusion errors in the LST (Borella et al. 2008)—The ratio between the number of words recalled that were not the last words in the sentences (intrusion errors) and the words recalled correctly in the LST was taken as an indicator of the efficacy of participants’ inhibitory control over the permanence of information in the WM.

#### Non-cognitive measures

Psychological Well-Being Questionnaire (WBQ) (De Beni et al. 2008). This is a 37-item questionnaire used in Italy to assess psychological well-being in adults and the elderly. It covers personal satisfaction, coping strategies, and emotion regulating skills. Participants were asked to rate their agreement with each of the 37 items using a 4-point Likert scale, ranging between 1 (not at all) and 4 (yes/often). Examples of items are: (i) “When I wake up in the morning, I am pleased with life and with myself”; (ii) “I feel that I am able to cope with difficult situations”; and (iii) “I can understand when someone is mad at me”. The overall score, considered as the dependent variable, is calculated as the sum of the scores for all items (maximum = 148). Higher scores indicate higher levels of psychological well-being. The reliability of the questionnaire was good (Cronbach’s α = 0.90).

State-Trait Anxiety Inventory (STAI Form Y_2_) (Zotti et al. 1985)—This is a 20-item self-report psychological inventory that measures trait anxiety—participants were asked to judge how frequently they felt as described by the items in everyday life, using a 4-point Likert scale (from 1 = almost never, to 4 = almost always). An example of the items is: "I get tired easily”. A higher score indicates higher levels of anxiety (maximum = 160). The reliability of the questionnaire was good (Cronbach’s α = 0.89).

Mindful Attention Awareness Scale (MAAS) (adapted from Brown and Ryan 2003).

The MAAS is a 15-item scale that reflects an ‘absence of mindfulness’ in everyday life. The items included, for instance: ‘I find it difficult to stay focused on what’s happening in the present,’ and ‘I do jobs or tasks automatically, without being aware of what I’m doing.’ Participants were asked to indicate how frequently they had each experience on a 6-point Likert scale ranging from 1 (almost always) to 6 (almost never). The total score was calculated by adding all the item scores. Higher scores indicated greater mindfulness. The reliability of the questionnaire was good (Cronbach’s α = 0.73).

#### Procedure

All participants filled in the informed consent than they tested individually during two sessions lasting approximately 90 min each. The vocabulary test, the SPMSQ (only for people > 65 years old), the WBQ, the MAAS, and the D2 were presented at the first session, and the SART, the LST, the Stroop color task, and the STAI Y_2_ at the second.

## Results

### SART accuracy

For each participant, we calculated the d-prime index from the percentage of correct hits and correct rejections.

The results showed no significant age-related differences regarding SART accuracy, *F*_(6,209)_ = 2.06, *p* = 0.06 (see Table 3).

**Table 3:** Descriptive statistics (M and SD) for the MW measures of interest by age group and MANOVA results

	Ages 20–29		Ages 30–39		Ages 40–49		Ages 50–59		Ages 60–69		Ages 70–79		Ages 80–89		*F*	*p*	η^2^
	N = 30		N = 30		N = 30		N = 30		N = 30		N = 30		N = 30				
	M	SD	M	SD	M	SD	M	M	SD	M	SD	M	SD	M			
*MW SART*																	
TUTs	0.22	0.26	0.24	0.27	0.21	0.28	0.20	0.31	0.10	0.16	0.07	0.13	0.05	0.12	3.70	.002	.10
SITUTs	0.06	0.12	0.08	0.12	0.06	0.11	0.04	0.08	0.05	0.08	0.01	0.03	0.02	0.04	2.04	.06	.06
EDs	0.09	0.14	0.11	0.13	0.10	0.19	0.10	0.20	0.02	0.05	0.03	0.08	0.00	0.02	3.49	.003	.09
TRIs	0.06	0.09	0.04	0.06	0.04	0.06	0.05	0.10	0.03	0.07	0.02	0.04	0.02	0.06	1.02	.41	.03
Not known	0.23	0.68	0.23	0.43	0.23	0.68	0.27	0.79	0.07	0.25	0.27	0.25	0.10	0.55	.078	.59	.02
Accuracy (d-prime)	4.57	0.19	4.64	0.01	4.62	0.12	4.59	0.22	4.60	0.21	4.56	0.26	4.42	0.56	2.06	.06	.06
*MW debriefing questionnaire*																	
self-perceived SITUTs	1.16	.90	.83	.69	1.00	.96	.92	.82	.68	.69	.60	.64	.54	.66	2.51	.02	.07
self-perceived EDs	1.73	1.05	1.73	1.28	1.50	1.38	1.63	1.38	.87	1.07	.90	1.16	.63	.93	4.65	< .001	.12
self-perceived TRIs	1.05	1.04	.85	.92	.75	.83	1.12	1.11	.85	1.17	1.40	1.32	1.63	1.43	2.43	.03	.08
Intentional TUTs	1.53	1.38	0.90	1.17	0.87	1.07	0.97	1.27	0.53	0.57	0.83	1.15	0.60	0.89	2.61	.02	.07
Unintentional TUTs	1.89	0.69	0.83	0.68	0.91	0.92	0.91	0.77	0.88	0.91	0.61	0.57	0.67	0.81	1.79	.10	.05

*SART* Sustained Attention to Response Task, *TUTs* task-unrelated thoughts, *SITUTs* thoughts or concerns about personal life and daydreams, *TRIs* task-related interference thoughts, *EDs* sensory perceptions/sensations or external distractions

### MW measures^2^

Participants reported that they had no difficulty in classifying the TUTs and that they did this accurately (mean scores > 5 for all age groups). There were also no significant age-related differences in the scores obtained by participants in the questionnaire assessing the variables: classification difficulty, *F*_(6,209)_ = 1.11, *p* = 0.36; classification accuracy, *F*_(6,209)_ = 1.21, *p* = 0.30.

ANOVAs were conducted to analyze age-related changes in each type of TUT during the SART and the debriefing questionnaire. For the SART, there was a significant main effect of group only for TUTs and EDs (see Table 3), with the oldest group (80- to 89-year-olds) reporting a lower frequency than participants in the 30- to 39-year-old age group (p = 0.034 and p = 0.037, respectively). For the debriefing questionnaire (see Table 3), there was a main effect of group only for the SITUTs, EDs and TRIs, with the oldest group significantly less distracted by external stimuli than participants aged 20–29 and 30–39 years (p = 0.009), or 50–59 years (p = 0.028).

To test the consistency of the off- and on-task TUT measurements, correlations were sought between the frequency of each type of TUT measured during the SART and the debriefing questionnaire. The results indicated that the measures across the two tasks correlated significantly for: total TUTs (r = 0.68, p < 0.01), SITUTs (r = 0.45, p < 0.001) and EDs (r = 0.55, p < 0.001), but not for TRIs (r = 0.07, p > 0.05).

Further, on analyzing the results of the debriefing questionnaire presented at the end of the SART, it emerged that younger adults reported a significantly higher frequency of intentional MW: the majority of the younger participants (20–29 years old) reported intentionally indulging in MW 30–40% of the time, while participants in the 60–69 (p = 0.012) and 80–89 (p = 0.026) age groups reported indulging in it < 20% of the time. There did not seem to be any age-related differences in unintentional MW.

### Correlation analyses

The correlation analyses were performed considering age, the rate of TUTs, SITUTs, EDs, and TRIs reported by participants during the SART, SART accuracy (d-prime index), and all the variables of interest (Table 4). The correlations were also analyzed, controlling for age.

**Table 4:** Correlations between measures of interest

	1	2	3	4	5	6	7	8	9	10	11	12	13	14	15
1. Age	–														
2. TUTs	− 0.34**	–	0.82**	0.75**	0.64**	− 0.01	0.06	0.26**	0.01	− 0.17*	− 0.06	0.08	0.14*	− 0.02	0.00
3. SITUTs	− 0.25**	0.84**	–	0.37**	0.23**	0.03	0.02	0.20**	0.05	0.12	− 0.05	0.07	0.13	− 0.04	0.09
4.EDs	− 0.37**	0.84**	0.64**	–	0.42**	− 0.09	0.09	0.16*	− 0.03	− 0.13	− 0.04	0.06	0.14*	− 0.03	− 0.15*
5.TRIs	− 0.20**	0.74**	0.63**	0.48**	–	0.02	0.05	0.21**	− 0.05	− 0.14*	− 0.08	0.02	− 0.01	0.03	0.04
6. SART accuracy	− 0.13	0.00	− 0.04	0.03	0.03	–	0.12	0.01	0.07	− 0.06	0.03	− 0.05	0.01	− 0.06	− 0.07
7. LST (correctly recalled)	− 0.61**	0.26**	0.23**	0.26**	0.15*	0.15*	–	0.39**	− 0.10	− 0.48**	− 0.11	0.02	0.02	− 0.04	− 0.05
8. D2 (correctly marked items)	− 0.68**	0.40**	0.30**	0.40**	0.28**	0.10	0.64**	–	− 0.18**	− 0.35**	− 0.18*	0.01	− 0.13	− 0.02	− 0.36
9. Color Stroop Interference Index	0.37**	− 0.10	− 0.07	− 0.12	− 0.10	− 0.02	− 0.31**	− 0.37**	–	0.31**	0.05	0.04	− 0.03	0.00	0.01
10. LST (intrusion)	0.38**	− 0.33**	− 0.27**	− 0.26*	− 0.29**	− 0.14*	− 0.67**	− 0.51**	0.28**	–	0.07	− 0.00	0.10	0.15*	0.12
11. SP-WBQ	0.27**	− 0.18*	− 0.08	− 0.18*	− 0.15*	− 0.05	− 0.25**	− 0.34**	0.16*	0.18*	–	0.52**	0.30**	− 0.50*	− 0.15*
12. SC-WBQ	0.05	0.05	0.10	0.05	− 0.02	− 0.07	− 0.03	− 0.04	0.05	0.06	0.49**	–	0.52**	− 0.42**	− 0.15*
13. EC-WBQ	0.14*	0.09	0.14*	0.09	− 0.01	− 0.01	− 0.05	− 0.20**	0.03	− 0.01	0.34**	0.48**	–	− 0.13	− 0.11
14. STAI	0.10	− 0.04	− 0.07	− 0.02	− 0.01	− 0.11	− 0.05	− 0.04	0.02	0.12	− 0.47**	− 0.37**	− 0.11	–	0.24**
15. MAAS	− 0.18*	0.09	− 0.06	0.09	− 0.06	0.07	0.07	0.11	− 0.07	0.04	− 0.19*	− 0.17*	− 0.15*	0.21**	–

*p < 0.05; **p < 0.01

*SART* Sustained Attention to Response Task, *TUTs* task-unrelated thoughts (log), *SITUTs* thoughts or concerns about personal life and daydreams, *TRIs* task-related interference thoughts, *EDs* sensory perceptions/sensations or external distractions, *LST* Listening Span Test, *WBQ* Well-Being Questionnaire, *SP-WBQ* Well-Being Questionnaire, personal satisfaction subscale, *CS-WBQ* Well-Being Questionnaire, coping strategies subscale, *EC-WBQ* Well-Being Questionnaire, emotional competence subscale, *STAI* State-Trait Anxiety Inventory, *MAAS* Mindful Attention Awareness Scale. Raw correlations are presented below the diagonal; correlations above the diagonal are controlled for age

As expected, increasing age coincided with a decrease in TUT frequency, and all its subcategories, a decline in performance on cognitive tasks, and an increase in the psychological well-being, personal satisfaction (PS-WBQ), and emotional competence (EC-WBQ) subscales, and in mindfulness (MAAS). No correlations were found between age and anxiety scores.

TUTs and their subcategories correlated positively with attention (D2) and WM (LST); in contrast with our expectations, TUTs correlated negatively with one of the inhibition measures used (intrusion errors in the LST). TUTs, TRIs, and EDs also correlated negatively with PS-WBQ.

Only the positive correlations between total TUTs (and all subcategories) and the negative correlation between TUTs, TRIs, and intrusion errors emerged after controlling for age. Likewise, after controlling for age, there was a positive correlation between TUTs, EDs, and EC-WBQ and a negative correlation between EDs and MAAS. The relationship between TUTs and these variables was further explored using path analysis.

### Regression analyses: effect of age

The nature of the effects of age on TUT^2^ and SITUT^2^ frequency across the adult life span was then tested. A hierarchical regression analysis was run to test the linear and non-linear trends of age (the quadratic age and the cubic term). The linear term for age explained 11% and 6% of the variance for the TUTs and SITUTs (*β* = -0.34, p < 0.001; and *β* = -0.24, p < 0.001), while the quadratic and cubic trends did not explain a significant part of the variance.

### Model estimation

Path analysis models (i.e., structural equation models using the variables observed) were further computed with the LISREL 8.7 statistical package (Jöreskog and Sörbom 1996) to test whether any effect of age on TUT and SITUT frequency was direct or mediated by working memory, attention, inhibition, and/or non-cognitive variables, all of which are thought to influence MW according to the theoretical framework adopted (see Smallwood and Schooler 2006; McVay and Kane 2010).

The dependent variable considered here was the frequency of TUTs^2^ (Model 1) and SITUTs^2^ (Model 2) reported during the SART, and the mediators included the cognitive measures, i.e., WM (words recalled in the LST), attention (items correctly marked in the D2 task), inhibition (the Stroop interference index and the proportion of intrusion errors in the LST), and the non-cognitive measures (scores on the three WBQ subscales, the STAI-X and the MAAS). Age was included as an independent variable.

An alternative model (Model b) was tested to see whether age mediated the relationship between the cognitive and non-cognitive variables (which became the dependent variables) on the one hand and the TUTs (Model 1b) or SITUTs (Model 2b), which became the independent variable.

**Fig. 1:**
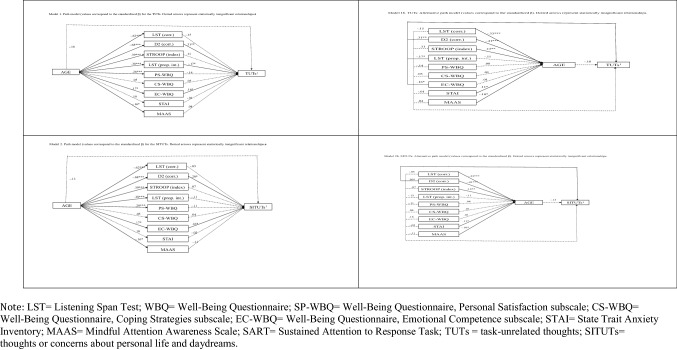
Path models for the TUTs and SITUTs

The path of Models 1 and 2 (see Fig. 1) showed good fit indices and explained 23% and 14% of the variance, respectively, in the reported frequency of TUTs and SITUTs. Models 1^3^ and 2 (on the TUTs and SITUTs) both identified a significant direct association between age and the following variables: words recalled in the LST; items correctly marked in the D2 task; Stroop Index; intrusion errors in the LST; the SP-WBQ; the EC-WBQ; and the MAAS. The direct effect of age did not reach statistical significance (see Fig. 1); its effect was mediated by the following direct significant relations: number of items correctly marked in the D2 task; the EC-WBQ; frequency of both TUTs and SITUTs; and proportion of intrusion errors, but only for the TUTs.

The alternative Model b did not have good fit indices for the TUTs and SITUTs, meaning that the relationship between TUTs or SITUTs and cognitive and non-cognitive variables was not significantly mediated by age (see Fig. 1 and Table 5).

**Table 5:** Path models for TUTs and SITUTs

	Variance explained	χ2	p	NNFI	CFI	RMSEA
*TUT*						
Model 1 (direct effect of age on cognitive and non-cognitive variables; indirect of age on TUTs; direct effect of cognitive and non-cognitive variables on TUTs)	23%	34.21	.062	.97	.99	.048
Model 1a as model 1, but only with significant relationships	19%	6.22	.044	.92	.97	.101
Model 1b (direct effect: TUTs on age; indirect effect of TUTs on cognitive variables; indirect effect: age on cognitive variables)	22%	0	1	1	1	0
*SITUTs*						
Model 2 (direct effect of age on cognitive and non-cognitive variables; indirect of age on SITUTs; direct effect of cognitive and non-cognitive variables on SITUTs)	14%	34.59	.057	.97	.99	.048
Model 2 a as model 1, but only with significant relationships	11%	4.84	.089	.94	.98	.083
Model 2 b (direct effect: SITUTs on age; indirect effect of SITUTs on cognitive variables; indirect effect: age on cognitive variables)	13%	0	1	1	1	0

The root-mean-square error of approximation (RMSEA), the non-normed fit index (NNFI), the comparative fit index (CFI), and a non-significant chi square (recommended by Schreiber et al. 2006) were considered as fit indices (Jöreskog and Sörbom 1993) to test the goodness of the model

## Discussion

The main goal of the present study was to assess the effects of age on MW, and particularly on TUT and SITUT frequency experienced across the adult life span (something never done before) up to very old age (89 years). We examined both overall TUTs and SITUTs because focusing on TUTs enabled us to compare our data with the majority of other MW studies, while SITUTs are more representative of distinctive core aspects of MW. A complementary goal was to explore the role of age and of cognitive and non-cognitive variables in explaining the frequency of TUTs and SITUTs, which have not been examined jointly before.

The overall results showed small age-related effects on MW variables (considering both TUTs and SITUTs). In particular, there were small, linear age-related effects on overall TUT frequency, due mainly to differences between the group over 80 years old and the 30- to 39-year-olds. A similar effect was found for SITUTs, for which the percentage of variance explained by age was again very small, and no significant differences emerged on comparing various age groups. There may be several reasons why these findings are in sharp contrast with a part of the MW literature (Jordão et al, 2019). For a start, although it is well known that there are different age-related changes in cognitive functioning in the third and fourth ages (e.g., Borella et al. 2008), the age ranges included in the so-called older age groups considered in the majority of MW studies vary considerably (from 60 to 90 years old) (see Jordão et al. 2019). The adult life span perspective (up to 89 years of age) taken here might therefore account for our results. On the other hand, they might be due to the SART used, which has proved a critical variable in whether or not age-related changes are found in MW, as suggested by Jordão et al. (2019). In fact, the present findings are in line with other studies on MW using a less demanding SART (see Table 4), in which older adults performed just as well as younger adults. A role for the type of SART is also supported by the results obtained for TRIs, which were more frequent in studies using complex tasks (see Maillet and Schacter 2016), while their frequency did not change significantly here because the SART was not very demanding. In short, the decrease in TUT frequency found here does not seem to involve SITUTs or TRIs (since their frequency did not differ significantly between various age groups).

A significant age-related effect was found for EDs (which always became less frequent in the oldest group), which could still indicate, however, that older people were better able to block the distractions elicited by external stimuli because they focused on the task more than younger people.

Though these aspects merits to be “directly” measured during the SART (and future studies should try to examine their impact on MW), the retrospective questionnaire we used pointed to age-related quantitative differences in the type of MW experienced. A significant decrease in intentional MW with aging (as found by Seli et al. 2017 in daily life situations) was, in fact, found, as if older adults (or the oldest, at least) would experience a proportional increase in the unintentional/less aware MW—typical of the second state of Cheyne and colleagues attentional engagement/disengagement model (Cheyne et al. 2009)—in which individuals are still able to carry out automated tasks (like the version of the SART used in our study) successfully, though they would make more mistakes in more complex tasks (e.g., McVay et al. 2013). Here again, the type of task used seems to crucially influence the effect of age on MW identified.

Path analyses results confirmed that it is not aging “per se*”* that leads to a reduction in the frequency of TUTs and SITUTs. The effect of age was, in fact, indirect and mediated by cognitive and non-cognitive variables that had a direct influence on TUTs and SITUTs. For both TUTs and SITUTs, the effect of age was mediated by attention and also emotional competence. The age-related changes in attentional resources thus seemed to explain the reduction in TUTs and SITUTs. This is consistent with the decoupling hypothesis, which postulates that people with fewer attentional resources tend to focus them all on the task at hand.

Intrusion errors had an unexpectedly direct negative association with TUTs, however. This would suggest that less efficient inhibitory mechanisms would prompt a decrease in TUT frequency, meaning that if information that is no longer relevant remains activated in the mental workspace (also in the form of external stimuli, as EDs) due to an impaired ability to resist proactive interference (measured here in terms of intrusion errors in the LST), it may saturate WM capacity (Borella et al. 2010; Robert et al. 2009), leaving fewer resources available for intentional MW. Though these findings seem to contrast with the McVay and Kane (2010) model, as suggested by Maillet and Schacter (2016), they do not necessarily argue against the executive control failures × current concerns view. They suggest instead that if older adults have fewer resources available for MW while completing a task or if they have fewer non-trivial concerns giving rise to MW than younger people, they may have less material to inhibit and therefore less competition between the task at hand and any SITUTs. So, as mentioned above, less demanding SART versions identifying small/null effects of age (see Table 1) may account for the less marked decrease in the MW frequency enabling participants with more attentional resources to focus only a part of them on completing the test, leaving some free to sustain MW processes. Our findings will therefore have to be replicated with more demanding tasks before we can draw any conclusions on this issue; they are, though, in line with those of Krawietz et al. (2012), who also found no relationship between MW and WM in aging. Nonetheless, since TUT frequency was found to mediate task performance (e.g., influencing recall or text comprehension, see Table 1), it would be interesting to see whether MW accounted for any effect of age on WM, in order to better capture the nature of the mechanisms behind their relationship.

We cannot rule out the possibility of the executive control failures × current concerns view explaining the trend of unintentional MW better than that of intentional MW. To shed more light on this aspect, it will be necessary to propose an intentional/unintentional categorization during the SART. In future studies, it could also be helpful to use an open-ended procedure, as suggested by Jordão et al. 2019. This would involve participants simply describing what is on their mind when probed (Weinstein et al. 2018), and could avoid making it too effortful to establish which category a thought belongs to (which would interrupt the normal attentional flow and raise the risk of misclassification).

The lack of a direct association between intrusion errors and SITUTs was unexpected, but may be due to the nature of the former. In fact, EDs (part of the TUTs) may relate to proactive interference, whereas SITUT frequency could be influenced only by other inhibitory functions not examined in this study. This is an issue that deserves to be better examined in future.

As for the non-cognitive variables, we found, as expected, that personal satisfaction, emotional competence, and mindfulness increased with age, but only emotional competence mediated the effect of age on TUT and SITUT frequency. To be more precise, the age-related increase in emotional competence explained an increase in TUT frequency. This result might seem at odds with the literature, but the emotional competence subscale of the WBQ pinpoints relationship-oriented people who are interested in others’ emotions and problems and who consequently have a greater tendency for TUTs. Clinical studies have shown that individuals interested in other people often become dissatisfied due to mood contagion leading to a cognitive burden in the listener (Kelly and McKillop 1996; Kowalski 2002). The insignificant role of personal satisfaction (one of the other subscales in the WBQ) in explaining TUT frequency could stem from the fact that this subscale measures a stable perception about personal life, whereas TUT frequency could be affected more by a transient positive affect than by characteristic well-being (see Frank et al. 2015).

Summing up, the small changes in MW with age across the adult life span and the fewer TUTs and SITUTs in older adults that we identified may be due to the type of SART used and to the influence of both cognitive and non-cognitive variables. Moreover, as our final debriefing questionnaire seemed to suggest—and in line with the findings of Gyurkovics et al. (2018), and Jordão et al. (2019)—there seems to be a change in the intentionality, or meta-awareness, of MW. Older adults have fewer attentional resources to devote to MW than younger adults, so they tend to focus all their attentional resources on the task at hand. They might also be more motivated than younger adults (see Seli et al. 2015a, b). These are mere speculations, however, as we did not administer any questionnaires that might support such a suggestion, and this represents one of the limitations of the present study. Future studies should also make an effort to address this issue directly by questioning participants about their intentionality during the SART and also to examine the role of processing speed in this intriguing phenomenon. This basic cognitive mechanism was not examined in the attentional task used in the present study, so caution is needed in interpreting the link between attention and MW in our sample. It is also worth bearing in mind that only a modest part of the variance in MW was explained by our models, so the debate on this issue is set to continue (Smallwood and Schooler 2006; McVay and Kane 2010). It would therefore be worth replicating the present results taking additional variables into consideration (i.e., personality traits, motivation, and interest) and manipulating the difficulty of the task (Jordão et al. 2019) to provide new insight on this fascinating topic.

In conclusion, the present findings, with the novelty of investigating age-related changes in MW across the adult life span and showing a sort of age-related resilience in MW with aging, are also a first attempt to shed light on the cognitive (attention) and other (emotional competence) variables capable of modulating MW over the whole adult life span.

## Footnotes

1 A preliminary analysis was conducted with the GPower program to ascertain the sample size required for the study. The results indicated an optimal number of 231 participants to obtain a mean effect size (f = 0.25), an alpha level of 0.05, and a power of 0.80. The study ultimately involved 210 participants (30 for each age group), thus coming very close to the optimal number, so the sample size was judged to be adequate.

2 A log transformation was applied to normalize the distribution of the TUTs and SITUTs.

3 We also ran Model 1, for both TUTs and SITUTs, after removing the non-significant variables, but the fit indices were not good (see Table 1, Model 1a and Model 2a). We therefore decided to run the alternative model using Model 1.
